# *Sho*-Based Kampo Medicine Combined With Assisted Reproductive Technology Is Effective for Refractory Infertility and Early Recurrent Miscarriage: A Case Report

**DOI:** 10.3389/fnut.2021.761199

**Published:** 2021-11-03

**Authors:** Hiromi Yoshida-Komiya, Masafumi Ami, Ryota Suganuma, Tadamichi Mitsuma

**Affiliations:** ^1^Center for Gender Specific Medicine, Fukushima Medical University, Fukushima, Japan; ^2^Ami Women's Clinic, Fukushima, Japan; ^3^Department of Obstetrics and Gynecology, Fukushima Medical University, School of Medicine, Fukushima, Japan; ^4^Department of Kampo Medicine, Aizu Medical Center, Fukushima Medical University, Fukushima, Japan

**Keywords:** Kampo, *Sho*, infertility, early recurrent miscarriage, assisted reproductive technology

## Abstract

Assisted reproductive technology (ART) is an effective treatment developed for infertile couples in the world. As a result, women suffering from infertility benefit from ART treatment. However, even when ART treatment is successfully performed, there are cases where conception is not achieved or maintained. Kampo medicine was originally developed in Japan, and *Sho* is the central part of Kampo concept. Although it is thought that Kampo medicine is useful for various women-specific symptoms in modern Japan, evidence is still lacking regarding the effectiveness of combination of *Sho*-based Kampo and Western medicine such as ART. In this article, we report a case of a patient with refractory infertility and early recurrent miscarriage (ERM) of unknown cause who successfully became pregnant with combination therapy of Kampo based on *Sho* and ART. The patient was a 34 year-old Japanese woman and had been treated with ART in a nearby clinic. In a 3 year period, she had undergone oocyte retrieval twice, frozen embryo transfer (FET) seven times and conceived twice. Since both conceptions ended in miscarriages and pregnancy could not be established thereafter, her clinic referred her to our hospital for Kampo treatment. As result of the diagnosis of her *Sho*-pattern, we chose Kampo medicine. Finally, she succeeded in conception 1.5 years after beginning treatment and was able to carry the fetus to term successfully. The current case showed that although our patient had been unable to give a birth after undergoing various western medical treatments for infertility, pregnancy was established and kept to term after addition of *Sho*-based Kampo treatment. Kampo medicine chosen by the *Sho*-patterns is useful for refractory infertility and ERM. It is important to note that examinations for evaluting the *Sho*-patterns are essential for selecting appropriate Kampo medicine. *Sho*-based Kampo leads to an increase in the effectiveness of ART treatment, and accumulation of evidence that clarifies *Sho*-pattern is required.

## Introduction

Women suffering from infertility due to ovarian insufficiency, tubal obstruction and even unknown causes have benefited from assisted reproductive technology (ART) treatment such as *in vitro* fertilization (IVF) and intracytoplasmic insemination (ICSI). The process of ART involves ovarian stimulation to produce multiple follicles, retrieval of the oocytes from the ovaries, oocyte fertilization and embryo incubation in the laboratory, and transfer of embryos into a women's uterus. Globally, the total number of ART cycles increased by almost 20% between 2011 and 2012, whereas pregnancy and delivery rates remained stable (1). In Japan, 454,893 treatment cycles were carried out in 2018, resulting in the birth of 56,979 neonates. The total number of both treatment cycles and neonates born in 2018 increased from 2017 (2). Although ART is now widely accepted as clinically effective for the treatment of many forms of infertility, some people remain unable to conceive due to poor reproductive function. In addition, the miscarriage rate per pregnancy established by ART in Japan is high, at 29.0% (2).

Kampo medicine originated around two thousand years ago in China. Herbal medicine grown in China was introduced to Japan in the Edo period (1603–1868), and from then it was uniquely developed as traditional Japanese medicine, named Kampo. Essentially, Kampo consists of a system of three dichotomies and three substances. The three dichotomies are as follows: Yin-You (yin-yang, positive-negative); Kyo-Jitsu (deficiency-excess); and Netsu-Kan (hot-cold). The three substance categories are qi, blood, and fluid. Qi is fundamental energy of life. Blood and fluid are similar with the common concepts of blood and bodily fluids. In Kampo, it is known that a well-balanced or non-deviated condition of the three dichotomies and three substance concepts leads to a healthy body (3). The International Statistical Classification of Disease and Related Health Problems, the 11^th^ version (ICD-11) approved in 2019 features new traditional medicine. A supplementary chapter of the classification includes symptomatology such as sign, symptoms, and unique findings using traditional medicine diagnostic methods to determine a pattern in traditional medicine that is known as *Sho* in Kampo (4). *Sho* is the central concept and essence of Kampo (5). The Kampo paradigm is completely different from that of modern Western medicine. Four specific diagnostic procedures are used for Kampo medicine, such as inspection, listening and smelling examination, abdominal examination and tongue examination. Especially, the abdominal examination is unique to Japan, and considered to be one of the most important approaches (6). Several studies have reported that the most suitable formula is chosen for a patient after determining *Sho* as a Kampo concept (3, 5, 7, 8). For example, Okita et al. (8) described their observations from a clinical trial that involved selected patients who met the criteria of Kakkonto-*Sho*, and demonstrated that it is more effective than conventional remedies.

In the present article, we report a case of a patient with refractory infertility and early recurrent miscarriage (ERM) who successfully became pregnant with a combination therapy of Kampo medicine, based on *Sho*, and ART. We describe that *Sho* is important for selecting the appropriate Kampo medicine and accumulating evidence to clarify the effectiveness of Kampo with ART.

## Case Presentation

The patient was a 34 year old Japanese woman who had a history of erythema exudative multiforme, and a family history of colorectal cancer in her mother and hypertension in her father. Menarche occurred in the patient when she was 13 years old, after which, she had constant menstrual irregularities. At the age of 30, she got married; she wanted to get pregnant immediately and was worried about her fertility because of her menstrual irregularity. She went to see an obstetrician and gynecologist in a nearby hospital. Although she had been treated with clomiphene or hormone therapy for 2 years at that hospital, pregnancy could not be achieved. She changed her doctors to an infertility specialist at a local clinic. At the first visit, physical examination showed that her height was 163.5 cm and weight was 52.4 kg. Other than a follicle-stimulating hormone (FSH) level of 2.5 mIU/mL (adult female follicular phase: 3.01~14.72 mIU/mL), hormone testing was normal, with a luteinizing hormone level of 2.3 mIU/mL (adult female follicular phase: 1.76–10.24 mIL/mL), an anti-Müllerian hormone level of 7.79 ng/ml, an estradiol level of 77.7 pg/mL (adult female follicular phase: 28.8–196.8 pg/mL), a progesterone level of 0.1 ng/mL (adult female follicular phase: <0.28 ng/mL), a testosterone level of 20.1 ng/dL (10.8–56.9 ng/dL), a prolactin level of 12.0 ng/mL (6.1–30.5 ng/mL), a thyroid-stimulating hormone level of 1.33 IU/mL (0.5–5.0 IU/mL), a free T3 level of 3.08 (2.3–4.0 pg/mL) and a free T4 level of 1.13 (0.9–1.7 ng/mL). Immunological blood test was negative for anti-nuclear antibody, anti-sperm antibody and anti- CL-B2GP1 antibody. Analysis of her husband's semen was within normal range. Both hysterosalpingography and hysteroscopy were almost normal, except for a slight bicornuate uterus.

Since transient hyperprolactinemia was observed after 3 years form the first visit of clinic, the patient was prescribed cabergoline for 3 months, after which the level of prolactin decreased. Even though her prolactin level was normalized, she remained unable to conceive. Therefore, the clinic doctor recommended that she undergo ovarian stimulation with recombinant FSH formulation. The first stimulation resulted in anovulation. The second led to an increase in the units of recombinant FSH formulation; as a result, the patient developed ovarian hyperstimulation syndrome. Because of her infertility of unknown origin and repeated failure in ovarian stimulation, she was recommended to try a form of ART such IVF and/or ICSI treatment, to which she agreed. Egg retrieval was performed twice and frozen embryo transfer was done seven times. Although she conceived at the third and seventh attempts, both conceptions resulted in miscarriage in the early pregnancy stage. She desired to improve her physical condition and decided to visit the Kampo clinic at our university hospital. The clinic doctor therefore referred her to our hospital for additional infertility treatment with Kampo, while still continuing ART at her local clinic.

**Figure 1:**
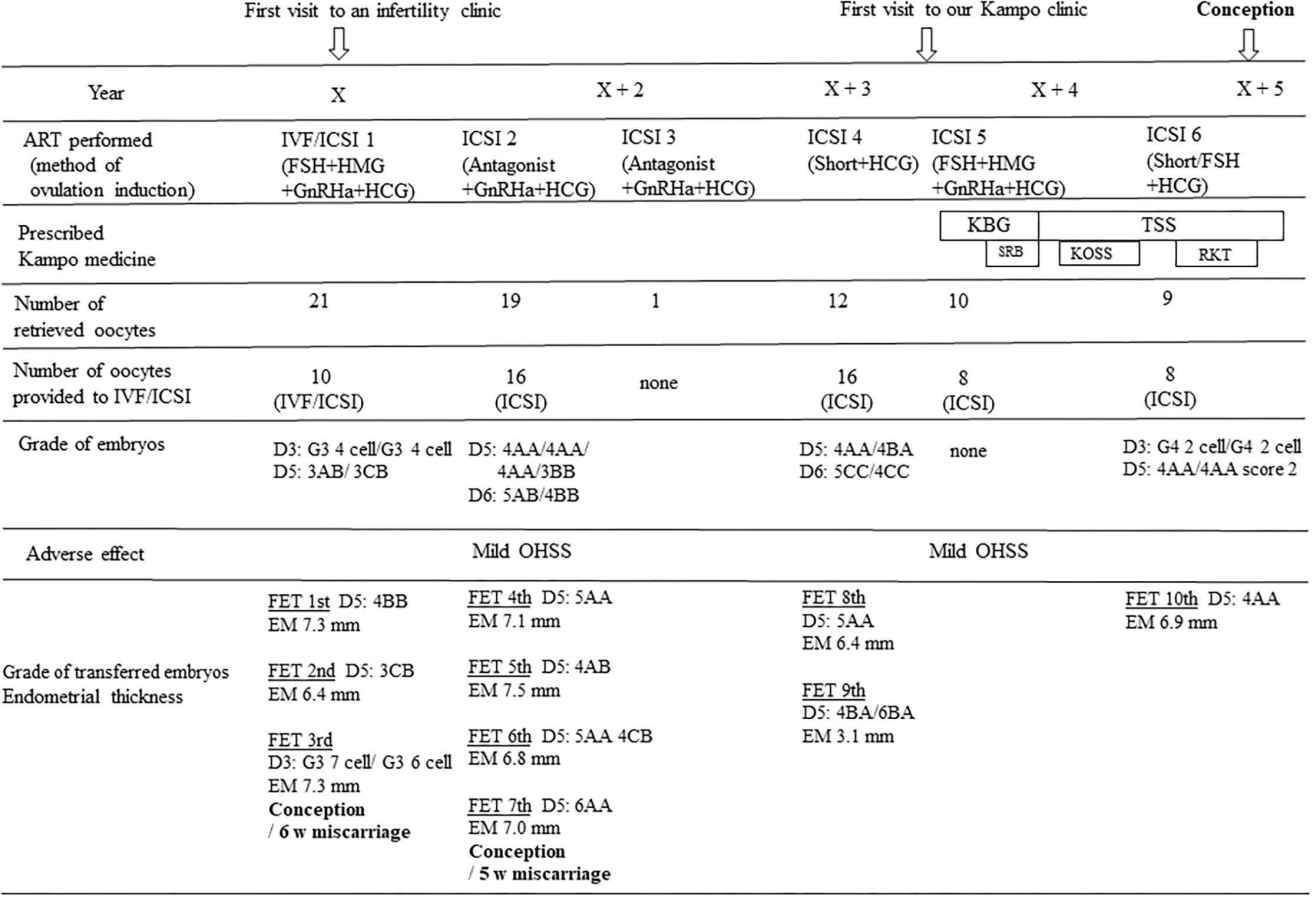
Time course of the treatment and results. There were no changes in number of oocytes retrieved for IVF/ICSI, grade of fertilized eggs and thickness of uterine endometrium before and after the addition of Kampo treatment to ART. The blastocyst score was determined according to Gardner's grading system, and early-stage embryos were evaluated with Veeck's criteria. IVF, *in vitro* fertilization; ICSI, intra cytoplasmic insemination; FSH, follicular stimulation hormone; HMG, human menopausal gonadotropin; GnRHa, gonadotropin releasing hormone agonist; HCG, human chorionic gonadotropin; Short, short protocol; KBG, Keishibukuryogan; TSS, Tokishakuyakusan; SRB, Saikokaryukotsuboreito; KOSS, Kososan; RKT, Rikkunshito; FET, frozen embryo transfer; OHSS, ovarian hyperstimulation syndrome; EM, endometrium. D3, day 3; D5, day 5; D6, day 6.

At the first visit, the patient complained of feeling hot. We performed examinations to determine her *Sho*-pattern. The abdominal examination and pulse examination revealed a strong abdomen and excessive pulse, respectively. We, therefore, made a diagnosis of yang and excess. In addition, we determined that she had blood stasis from the findings such as para-umbilical tenderness, resistance, and lower abdominal resistance, as well as a dark reddish color and sublingual vein distension in the tongue inspection results. We chose a Kampo medicine, keishibukuryogan (KBG, decoction, three times per day before meals), which improves blood stasis, and prescribed for 2 weeks. At the second visit after 2 weeks, she complained of mental instability. Since Kampo finding showed epigastric discomfort and resistance, hypochondriac discomfort and distension, and brisk epigastric aortic pulsation were observed, we considered it to be qi stagnation and added saikokaryukotsuboreito (decoction, three times per day before meals) to KBG to improve her qi. Her hot feeling and mental instability disappeared for a while. After 6 months, she had the feeling of cold extremities, leg edema and tiredness. She described busyness of her work and depressed mood due to the repeated failure of ART. Examinations such as abdominal palpation, tongue inspection and pulse sign revealed a gradual change in the patient's *Sho* pattern from yang and excess to yin and deficiency pattern. Abdominal strength weakened, para-umbilical tenderness was observed, and splashing sound in the epigastric region was noted. Tongue inspection showed a slightly swollen tongue that was pale pinkish in color. Her pulse was weak and floating. We changed the KBG prescription to tokishakuyakusan (TSS, decoction, three times per day before meals) which is used for blood deficiency and fluid disturbance. As qi disturbance was observed during the TSS treatment, kososan (TJ-70, 7.5 g/day, three times per day before meals, TSUMURA & CO, Ltd., Tokyo, Japan) for qi stagnation and rikkunshito (TJ-43, 7.5 g/day, three times per day before meals, TSUMURA & CO, Ltd., Tokyo, Japan) for qi deficiency were added to TSS. Gradually, her feeling of cold extremities, leg edema and tiredness improved. Finally, the patient succeeded in conception after 1.5 years from the beginning of Kampo treatment. At 38 weeks of gestation, she gave birth to a boy by cesarean section because of breech presentation. The newborn had Apgar scores of 8 at 1 min and 7 at 5 min. Her clinical time course from the beginning of ART to conception is shown Figure 1.

## Discussion

In the present article, we reported a case of a patient with refractory infertility and ERM who successfully became pregnant after Kampo treatment based on *Sho*. The patient had irregular menstruation before marriage. Therefore, she decided to go into fertility treatment immediately after getting married. Finally, she achieved conception and maintained pregnancy 7 years after beginning infertility treatment. We think that it is uncertain whether the addition of Kampo treatment to ART had a direct effect on the conception and pregnancy. However, until Kampo medicine treatment was started, the patient could not give a birth for 5.5 years, even though she was treated with various western treatments. She mentioned that she felt her physical and mental conditions had improved after starting a Kampo medicine. Therefore, we believe that additional Kampo medicine to ART treatment was effective for our patient, even though it is uncertain whether it had direct effects on the reproductive organs.

There are several reports, including systematic reviews, on the effects of traditional Chinese medicine (TCM) for infertile women (9–13). Most of these demonstrated the effectiveness and safety of TCM, although a small number of trials did not show effectiveness. Lee et al. performed a systematic review in 2015 and showed a better clinical pregnancy rate (RR 1.74, 95% CI 1.56–1.94) with herbal medicine (13). For infertile women undergoing IVF, Cao et al. (9) reported significant effects of TCM on clinical pregnancy rate (OR 2.04, 95% CI 1.67–2.49) and ongoing pregnancy rate (OR 1.91, 95% CI 1.17–3.10). Although these two studies demonstrated that TCM is effective for infertile women with or without the use of Western medicine, there were several differences between them such as contents of medicine, duration of medication and method of administration. Therefore, at present, there are some limitations with TCM treatment for infertility, especially regarding a lack of uniform standards.

Alternatively, Kampo is a unique, traditional Japanese herbal medicine, with *Sho* in its central concept (5). In the present case, at the first visit to our hospital, the patient was diagnosed as yang and excess pattern, and blood stasis. According to the diagnosis, we prescribed KBG. Since her pattern changed from yang to yin pattern after 6 months, we changed her Kampo medicine from KBG to TSS. Both KBG and TSS are typically prescribed for the treatment of menstruation-related symptoms such as irregularities, premenstrual syndrome, dysmenorrhea and menopausal syndrome. As shown in Table 1, KBG is used for yang and blood stasis, and TSS is used for yin, blood deficiency, fluid disturbance and blood stasis. The important points are that the *Sho*-pattern needs to be carefully followed during the infertility treatment, and the change of its pattern should not be missed.

**Table 1:** Prescriptions and components of KBG and TSS.

	**Keishibukuryogan (KBG)**	**Tokishakuyakusan (TSS)**
Components	(1) Keihi = Cinnamon Bark 4 g (2) Bukuryo = Poria Sclerotium 4 g (3) Shakuyaku = Peony Root 4 g (4) Botanpi = Moutan Bark 4 g (5) Tounin = Peach Kernel 4 g	(1) Toki = Angelica Root 3 g (2) Senkyu = Cnidium Rhizome 3 g (3) Shakuyaku = Peony Root 4 g (4) Bukuryo = Poria Sclerotium 4 g (5) Byakujyutu = Atractylodes Rhizome 4 g (6) Takusya = Alisma Tuber 4 g
*Sho*	yang and excess, blood stasis	ying and deficiency, fluid disturbance, blood deficiency, blood stasis
Target group	hot flashes, tenderness in para-umbilical area	cold extremities, edema, dizziness, splashing sound in epigastric region, tenderness in para-umbilical area
Indications	menstrual irregularity, dysmenorrhea, menopausal syndrome	anemia, menstrual irregularity, dysmenorrhea, menopausal syndrome, infertility, various symptoms during pregnancy such as edema, recurrent miscarriage, and abdominal pain

Previous studies have reported that several Kampo medicines were effective in women with infertility. Ushiroyama demonstrated clinical usefulness of Unkei-to in anovulatory and/or infertile women (14). It is suggested that Unkei-to targets at the hypothalamus and the pituitary glands and that its mechanism involves adjusting the gonadotropin level *in vivo* to a physiologically appropriate level. Otani et al. reported the usefulness of hachimijiogan for treatment of hyperprolactinemic infertile women with a pituitary microadenoma (15). In their report, although the patient was resistant to bromocriptine, she succeeded in having a normal pregnancy and delivery with hachimijiogan. Usuki et al. (16) showed that TSS improved luteal insufficiency in women but did not affect the hormonal levels with normal menstrual cycles. As a molecular mechanism for ovarian function, it has been reported that TSS stimulated progesterone and estradiol-17 beta in rat granulosa cells (17). Regarding the uterus, Terawaki et al. (18) reported that the ameliorating effects of TSS in a rat model of implantation failure may involve the alleviation of decreased leukemia inhibitory factor production derived from the endometrial gland, and decidualization dysfunction. Certainly, in the current case, we suspect that TSS impacted not only implantation but also miscarriage prevention.

It is not clear why the *Sho* pattern in our patient changed 6 months after starting treatment with KBG. Imai et al. (19) reported that lower educational background, longer duration of infertility (>2 years), being non-permanent worker, harassment experience in the workplace, and lack of support within one's company were identified as risk factors for stress after initiating infertility treatment. It is possible that infertility itself and infertility treatment are associated with increased distress. Especially, insurance medical treatment is currently not available for ART treatment in Japan, and continuing ART treatment for long periods of time can be stressful from an economical perspective. We suppose that the patient in the current report might have felt depressed and stressed due to recurrent unsuccessful ART results.

This case report had a few limitations. First, we showed only one case. The repeatability of KBG and TSS on infertility should be warranted through rigorously designed clinical trials based on *Sho*. Second, the different mechanisms of actions of KBG and TSS remain unclear. Further study will be needed for characterizing the mechanism for whole body and reproductive organs such as uterus and ovary. Third, although *Sho* in Kampo and *Zheng* in TCM were derived from the same word, they have acquired different meanings (7, 20–22). In this case report, it should be noted that we described *Sho* in Kampo. On the other hand, there are strengths in this report. One is, since we are specialized in Kampo medicine, we could accurately diagnose *Sho* of the patient. The other is, we could coordinate medical treatment performed by Kampo medicine specialists and a doctor specialized in reproductive medicine in the patient.

In this case, in particular, we emphasized the value of *Sho* evaluation in the intractable condition and could show how *Sho* changed in the treatment course over the years. *Sho* is an indicator of evaluation for not only individual organs, but also general condition. Kampo medicine contributed to the correction of her systemic condition. As a result of microenvironment improvement, functional recovery of the reproductive organs such as uterus and/or ovary might have been achieved.

## Conclusions

The combination of Kampo with Western medicine such as ART for infertility treatment is considered effective for achieving conception in women with refractory infertility and ERM. In addition, it is important to note that evaluation of the *Sho*-patterns, via examinations such as abdominal palpation, tongue inspection and pulse signs, is essential for selecting the appropriate Kampo medicine. If medical doctors prescribing Kampo can diagnose the patient's *Sho*, the efficiency of treatment might increase. At present, evidence that clarifies *Sho*-patterns in Kampo medicine is not enough concerning its effectiveness in combination with ART. Further studies are needed to accumulate sufficient evidence.

## Data Availability Statement

The original contributions presented in the study are included in the article/supplementary material, further inquiries can be directed to the corresponding authors.

## Ethics Statement

Written informed consent was obtained from the participant for the publication of this case report.

## Author Contributions

All authors provided contributions to the conception, design, drafting, and critical revision of the study. All authors have read the manuscript and approved it for submission for publication.

## Conflict of Interest

TM received a grant from TSUMURA & Co., and lecture fee from TSUMURA & Co., and Kotaro Pharmaceutical Co., Ltd. The funder was not involved in the study design, collection, analysis, interpretation of data, the writing of this article or the decision to submit it for publication. The remaining authors declare that the research was conducted in the absence of any commercial or financial relationships that could be construed as a potential conflict of interest.

## Publisher's Note

All claims expressed in this article are solely those of the authors and do not necessarily represent those of their affiliated organizations, or those of the publisher, the editors and the reviewers. Any product that may be evaluated in this article, or claim that may be made by its manufacturer, is not guaranteed or endorsed by the publisher.
